# Co-movement and Granger causality between Bitcoin and M2, inflation and economic policy uncertainty: evidence from the U.K. and Japan

**DOI:** 10.1016/j.heliyon.2022.e11178

**Published:** 2022-10-24

**Authors:** Provash Kumer Sarker, Lei Wang

**Affiliations:** a Institute of Central China Development, Wuhan University, 430072, Hubei, China; b Deputy Director, Bangladesh Bank, Dhaka-1000, Bangladesh

**Keywords:** Bitcoin, M2, EPU, Inflation, Wavelets, Nonlinear causality, Toda-Yamamoto causality

## Abstract

This study aims to investigate the co-movement and Granger causality between Bitcoin prices (BTC) and M2 (cash, demand, and time deposits), inflation, and economic policy uncertainty (EPU) in the U.K. and Japan. It uses monthly data from 31 July 2010 to 30 August 2020 and employs the wavelet coherence method, Toda-Yamamoto, and nonlinear Granger-causality tests. The empirical results show that (i) Bitcoin prices influence M2 and interact with inflation and EPU. In the short term, inflation affects Bitcoin price positively, supporting Bitcoin as an inflation hedged instrument in Japan. Both in Japan and the U.K., the short-term effects of M2 on Bitcoin prices are negative, while EPU's effects on Bitcoin prices are positive, (ii) a bidirectional Toda-Yamamoto Granger causality exists between Bitcoin prices, inflation, and EPU and confirms that M2 affects Bitcoin prices, (iii) a nonlinear bidirectional causality exists between Bitcoin prices and inflation. While Bitcoin prices Granger cause M2 in the U.K. and Japan, inflation shows a nonlinear Granger causality with EPU in Japan. These findings help investors make investment decisions while considering the effects of M2, inflation, and EPU, and monetary authorities and policymakers make policies involving Bitcoin.

## Highlights

• Bitcoin prices affect M2 (cash, demand, and time deposits) and inflation in the U.K. and Japan.
• Economic policy uncertainty (EPU) positively affects Bitcoin in the short term.
• Inflation nonlinearly Granger causes EPU in Japan.
• M2 also Granger causes Bitcoin in the U.K.

## 1 Introduction

Bitcoin is becoming a singularly popular digital asset. Bitcoin is an electronic currency and independent of regulation (Nakamoto, 2008). Recently, the debates about Bitcoin have intensified thanks to its extreme price volatility, higher returns, and hedging properties. Its growing adoption as a currency and a financial asset has fuelled profound concerns. As the additive use of Bitcoin generates additional demand for it, Bitcoin can significantly get along with the common platform of fiat currency and substitute the latter. Besides, high uncertainties such as exchange rate volatility, currency crises, and inflationary pressures in fiat currency redirect investors to Bitcoin. Wang et al., 2019a, Wang et al., 2019b report that Turkish investors shifted their investments to cryptocurrency during Turkey's currency crises. Recently, Bitcoin showed a meteoric rise as institutional investors, investment banks, and fund managers, such as Square, JPMorgan, Goldman Sachs, and Black Rock, continue to support the cryptocurrency by including the asset and related companies in their portfolios. The U.S. regulator approved the first Bitcoin ETF on New York Stock Exchange. Institutional investors are increasingly buying up Bitcoin. Notably, around US$6,300 million investment in Bitcoin recently by two U.S. giants (Tesla and MicroStrategy Inc.) and US$9,600 million by other 18 listed companies, increasing acceptance and involving long-term implications.

Institutional investors' recent uptick in Bitcoin is a novel advancement that can add Bitcoin to the regular finance and monetary system. For example, The Central Reserve Bank of El Salvador has declared Bitcoin as its fiat currency. Although central banks regulate the price levels of traditional currencies through monetary policy, no policymakers or monetary authorities regulate cryptocurrencies (Schilling and Uhlig, 2019). Thus, abnormal price fluctuations and incomparably high Bitcoin returns attract investors who see cryptocurrencies as new financial assets to invest in Bitcoin (Celeste et al., 2020). Besides, Gandal et al. (2018) argue that as an electronic currency, Bitcoin can destabilize fiat money and traditional payment options. Therefore, Bitcoin can be a systematic risk, which necessitates an examination of its financial and macroeconomic consequences. Hence, considering Bitcoin's monetary policy and financial market implications, the examination of the co-movement of Bitcoin prices (BTC) with M2, inflation, and EPU seems warranted.

However, to date, most studies in the existing literature consider Bitcoin price and volatility (Ciaian et al., 2015; Katsiampa, 2017; Phillips and Gorse, 2018; Goczek and Skliarov, 2019; Takaishi, 2021; Wu et al., 2022), the linkages between Bitcoin and financial markets (Baur et al., 2018; Shahzad et al., 2020; Matkovskyy et al., 2020; Zhang et al., 2021a, Zhang et al., 2021b), the relationship between fiat currency and Bitcoin (Jin et al., 2021). However, only several studies examine the connection between EPU and Bitcoin (Demir et al., 2018; Wang et al., 2019a, Wang et al., 2019b, Wang et al., 2020a, Wang et al., 2020b Mokni, 2021). Mokni et al. (2020) examine the effects of EPU on the Bitcoin-US stock relationship and find a negative effect due to the Bitcoin crash in December 2017. The authors assert that adding Bitcoin to the U.S. stock portfolio helps diversify the risk. Zhang et al., 2021a, Zhang et al., 2021b investigate the relationship between EPU and corporate risk-taking. Hernandez et al. (2021) examine the short- and long-term effects of EPU on Bitcoin, gold, and the implied U.S. stock market volatility (VIX) and find that EPU significantly negatively affects Bitcoin over short horizons. Focusing on the risk transmission between Bitcoin and traditional financial assets during the COVID-19 era, Elsayed et al. (2022) find that EPU is the only global factor that causes higher volatility in Bitcoin prices.

Nonetheless, much less evidence is available on the interactions between Bitcoin and M2 and inflation. Notably, Wang et al. (2022) recently found that Bitcoin affects M2 and inflation in the U.S., however, the authors did not show any evidence from other advanced countries like the U.K. and Japan, which would provide comprehensive insights for Bitcoin investors across the world. This study considers Japan and the U.K. for two specific reasons. First, the growing literature lacks studies on the relationship between Bitcoin and M2, EPU, and CPI in the context of the U.K. and Japan. In a recent study, Wang et al. (2022) examine the relationship between Bitcoin and money supply, EPU, and inflation in the case of the U.S. only. Second, Bitcoin is used as' just-like-money' in Japan, implicating various economic and financial consequences. Besides, Japan has one of the largest Bitcoin crypto exchanges in the world, i.e., bitFlyer. The monetary policy of Japan directly interacts with Bitcoin as it is a fiat-equivalent currency instrument in Japan. People in Japan can use Bitcoin as a payment instrument and digital financial assets, which influences the velocity of the Japanese Yen. In the U.K., Bitcoin is legal and increasingly popular among investors and users. Crypto exchanges such as Gemini, eToro, or Coinbase facilitate daily Bitcoin trade and transactions in the U.K. Moreover, the increasing adoption of Bitcoin by more than 214 merchants and 81 crypto-ATMs in the urban U.K. and 260,000 stores in Japan (Helms, 2022) triggers the concern for potential financial and macroeconomic effects, which need investigation. Thus, the present study considers the U.K. and Japan to examine the co-movement and causality, employing three different methods. Understanding the dynamic co-movements of Bitcoin has a tactical understanding for upgrading policies and fine-tuning investment decisions. Thus, this research investigates co-movement and Toda-Yamamoto and nonlinear causality between BTC and M2, inflation, and EPU.

The novelty of the study is threefold. First, this study is the first to investigate the dynamic co-movements and Granger causality between Bitcoin prices and M2 and inflation in the U.K. and Japan. Second, this study employs three different methods, i.e., wavelet coherence, which captures the lead-lag relationship among the variables, linear (Toda-Yamamoto), and nonlinear Granger causality tests on BTC, inflation, and EPU in the U.K. and Japan, capturing the linear and nonlinear relationships**.** In doing so, this paper contributes significantly compared to the previous studies (e.g., Bouri et al., 2017; Narayan et al., 2019; Mokni, 2021; Blau et al., 2021; Li et al., 2022), which use only linear methods. The current study applies linear, nonlinear, and wavelet methods to examine the co-movement and Granger causality between the BTC, M2, inflation, and EPU. Besides, most studies focus on a single economy, i.e., the U.S., and economic indicators such as monetary policy and fed rates, while the current study considers M2 in the context of two advanced countries, i.e., the U.K. and Japan, where Bitcoin is legal and frequent. Third, besides complementing the existing literature on inflation and Bitcoin (Conlon et al., 2021; Choi and Shin, 2022; Marmora, 2022), this study's novel findings add that EPU positively leads BTC in the U.K. and Japan. Moreover, the current study finds a nonlinear bidirectional causality between BTC and inflation, while BTC can predict M2 in both countries. The study's findings involve policy implications of regulating Bitcoin and M2 for central banks in the U.K. and Japan.

Regulatory policies on Bitcoin can immensely influence the investment and trading behavior of investors. Different countries and regulatory authorities manifest mixed views on cryptocurrencies. Specifically, France, Germany, and the U.K. are yet watchfully observing and evaluating the legal and regulatory framework for cryptocurrencies. Thus, these findings are useful for the individual, and institutional investors to make investment decisions and provide valuable information to central banks regarding Bitcoin's potential integration as a national currency or legal adoption as a financial asset within the global financial domain.

The rest of the paper is as follows. Section 2 revisits the existing literature on Bitcoin prices and cryptocurrencies, while section 3 formulates the research hypothesis and presents the economic rationale behind the interrelationship between Bitcoin prices, M2, inflation, and EPU. Section 4 describes the data and econometric methods. Section 5 shows and interprets the empirical results from the wavelet coherence, linear and nonlinear Granger causality tests, and post-estimation tests. Section 6 concludes with policy implications.

## 2 Related studies

This section revisits the recent academic literature on Bitcoin to contextualize the study and link Bitcoin to the M2, inflation, and EPU. Specifically, we review the contemporary literature from three broad perspectives.

### 2.1 Bitcoin as a financial asset and hedging instrument

Many academic studies classify Bitcoin as a financial asset, e.g., an investment vehicle or an alternative investment (see, Böhme et al., 2015; Bianchi, 2020; White et al., 2020; Béjaoui et al., 2021; Diaconaşu et al., 2022). Kristoufek (2015) finds that Bitcoin forms a distinct asset property similar to financial assets. Dyhrberg (2016) classifies Bitcoin as an electronic financial investment and shows that Federal fund rates can affect Bitcoin prices. Gkillas and Longin (2019) consider Bitcoin as gold and examine the potential benefits in harnessing returns.

Other studies consider Bitcoin a hedging instrument against inflation, gold prices, oil prices, and overall uncertainty, yet they show mixed results. For example, some studies claim Bitcoin is a hedging investment for uncertainty (Bouri et al., 2017), an instrument for portfolio diversification, or a hedging asset (Feng et al., 2018; Su et al., 2020). Applying the wavelet method, Bouri et al. (2017) found that Bitcoin can defend against uncertainty, which was later questioned by (Demir et al., 2018), who assert that EPU can negatively affect Bitcoin prices. In addition, Gozgor et al. (2019) employ the wavelet methods and find that Bitcoin investment can swap against inflationary expectations. The authors presume that a diversified Bitcoin portfolio can help defend against unforeseen risks and confirm the safe haven properties of Bitcoin against inflation. Wang et al., 2019a, Wang et al., 2019b explore whether Bitcoin can be used either as a hedging or a safe haven financial asset and find that Bitcoin can be utilized as a hedging instrument against shares, bonds, and money market tools. Wang et al., 2020a, Wang et al., 2020b explore the hedge and safe haven properties of stablecoins against traditional currencies and find that the safe haven property of stablecoins changes across market conditions. Mroua et al. (2022) claim that investors can add Bitcoin as it improves the performance of a traditional financial portfolio, however, the authors did not indicate whether Bitcoin is shielded from inflation and uncertainty. However, Su et al. (2022), applying the rolling-window Granger causality, find that fear sentiment adversely affects Bitcoin prices and, thus, Bitcoin cannot be a safe haven in fear sentiment conditions. This challenges the safe haven properties of Bitcoin because the fear of Bitcoin's price crash drives investors to disinvest in Bitcoin. Choi and Shin (2022) explore the hedging properties of Bitcoin against inflation and uncertainty and find that Bitcoin can shield itself from inflation, but its prices decline under increased uncertainty, which implies Bitcoin is not a safe haven. The present study's research findings extend the above-cited literature by providing advanced knowledge on linear and nonlinear causality between Bitcoin prices and economic variables and their dynamic interdependence over scales and frequency and causality. This is important as market participants in the cryptocurrency markets have heterogeneous investment horizons ranging from days (for day traders) to years (for institutional traders).

### 2.2 Bitcoin as an alternative currency

Bitcoin is used as a payment instrument in many countries. Due to its lower transaction costs and anonymity, Bitcoin is growing increasingly popular among users. Several empirical studies consider Bitcoin a medium of exchange (Janson and Karoubi, 2021; Rogojanu and Badea, 2014; Dwyer, 2015; Baur et al., 2018; Marmora, 2022). Compared to fiat currency, Bitcoin possesses very low transaction costs, no inflation, and anonymity (Ciaian et al., 2016; Kim, 2017), perfecting Bitcoin as a more payment-friendly exchange than fiat currencies. Many advanced and emerging economies now allow Bitcoin as a payment instrument, while some cryptocurrency exchanges are now officially established, allowing investors to trade Bitcoins against fiat currencies (Böhme et al., 2015). Further, Hazlett and Luther (2020) find that Bitcoin can be a substitute for fiat currencies, signifying that the replacement effects may destabilize the M2. Therefore, the cumulative use of Bitcoin in digital payments, investments, and remittances can reduce the velocity of fiat currency. On the other hand, Levulytė and Šapkauskienė (2021) show that the movements of fiat currency affect cryptocurrency markets.

Above mentioned growing literature examines different financial and economic properties of Bitcoin. However, the financial and monetary implications of using Bitcoin as a fiat currency and financial asset are unexplored (Wang et al., 2022). To the best of our knowledge, no studies in the existing literature investigate the co-movement and causality of Bitcoin with M2, inflation, and EPU in the context of the U.K. and Japan. This study fills this research gap by establishing the economic linkages between Bitcoin, M2, inflation, and EPU and then investigating the dynamic co-movement and Granger causality between them using wavelet coherence and linear and nonlinear causality methods. The current study provides the first-ever evidence of Bitcoin affecting the M2 in the U.K. and Japan.

## 3 Research hypothesis

The theoretical foundation establishing an economic relationship between Bitcoin prices, M2, inflation, and EPU concerns the substitution effects that can channel via dual transmission paths: (1) the exchange rate path and (2) the payment path. The present study provides the following three hypotheses expounding how the substitution impacts may occur through the paths mentioned above.

Hypothesis 1 Bitcoin prices affect M2.

Bitcoin price as a digital financial asset and payment instrument provides higher returns and ease of transition to investors and users. The study assumes that adopting Bitcoin as an alternative currency and a financial asset in an economy can replace M2. The replacement effects can occur through exchange rate and payment channels.

Bitcoin can substitute traditional currencies and affect M2 by altering the velocity of traditional money and decreasing the demand for fiat currency. The velocity is denoted by the Equation of Exchange, i.e., V=P⁎T/M, where $V_{t}$ indicates the velocity of money, P represents the price, T is the aggregate values of transactions, and M is the money-in-circulation. The growing use of Bitcoin replacing fiat currency can decrease the frequency and value of T, which, in return, decreases V, holding M and P unchanged. Increasing adoption of Bitcoin by more than 214 merchants in the urban U.K. and 260,000 stores in Japan (Helms, 2022) and Bitcoin ATMs may affect (decrease) the velocity of the Pound sterling (GBP) and Yen. Thus, Bitcoin can significantly involve currency substitution (Schilling and Uhlig, 2019; Marmora, 2021), which can affect M2.

Besides, as a financial asset, Bitcoin can decrease the demand for fiat currency, involving a substitution effect. Thus, the present study considers the demand for money equation to clarify the effect transmission. The equation is $M_{d}=P⁎L(r,Y)$, where $M_{d}$ denotes money demanded, P is the price, and the term L(r,Y) indicates cash holding preference, r is the nominal interest rate, and Y is the output. When r decreases, investors feel motivated to invest their savings in financial assets with higher interest rates. In such an event, Bitcoin's extraordinary returns attract more investors to invest in Bitcoin. Accordingly, a lower r, all else being fixed, reduces demand for fiat currency and increases investment in Bitcoin. Thus, Bitcoin as a financial investment vehicle may alter the M2. Because the use of Bitcoin is increasing globally, this can lead to a global phenomenon through which fiat currencies are gradually substituted with cryptocurrencies, and international financial institutions are derived to innovate (Seetharaman et al., 2017; Marmora, 2021). Bordo and Jonung (1981) examine the velocity of broad money (M2) in five developed economies and show that the velocity of money reduces with monetization. Bitcoin is a financial innovation (Li et al., 2021), and financial innovation substantially affects the velocity of money, eventually affecting the M2 (Lu et al., 2016). Considering Bitcoin, a store of value and payment instrument, Narayan et al. (2019) show that it negatively affects money velocity by substituting money. Accordingly, the use of Bitcoin as an alternative to fiat currency will decrease the velocity of money in circulation. Based on the above discussion, the study hypothesizes that Bitcoin prices influence M2 and tests the relationship in the context of the U.K. and Japan.

Hypothesis 2 Changes in inflation affect Bitcoin prices.

Inflation can affect financial investments through a risk-return trade-off. Inflation decreases fiat currency's purchasing power; thus, investors always prefer financial assets safe from inflationary pressure. When investors expect inflation to rise, they may invest in financial assets such as Bitcoin, which will create additional demand for Bitcoin, leading to a price increase. In general, uncertainty in fiat currency caused by inflation and currency crises creates additional demand for Bitcoin, which could eventually increase Bitcoin prices (Jin et al., 2021). Bitcoin is perceived to hedge against inflation. Bitcoin is exchangeable in the commodity and currency market (Ciaian et al., 2015). Inflation depreciates the value of deposits in fiat currencies over time, which motivates individuals to invest in inflation-hedged assets, such as Bitcoin. Hence, higher inflation rates demotivate investors to put their savings at financial institutions and attract them to invest in an inflation-hedged asset. As a result, higher inflation rates increase the demand for Bitcoin. Bitcoin directly reacts to inflation expectations (Marmora, 2021). A recent study shows that cryptocurrencies are positively related to inflation (Conlon et al., 2021), and Bitcoin, in particular, has a unidirectional causal relationship with inflation (Blau et al., 2021). Choi and Shin (2022) examine whether Bitcoin can hedge against inflation and reveal that Bitcoin prices appreciate under inflationary pressure. In general, an increase in demand for Bitcoin devalues fiat currency. Thus, a link between Bitcoin and inflation is established through substitution and payment implications, and therefore, the present study hypothesizes that changes in inflation significantly affect Bitcoin prices through the demand function.

Hypothesis 3 Economic policy uncertainty news affects Bitcoin prices.

The world economy is a dynamic state of economic policy regulations. A subtle change in economic policy uncertainty may drastically affect financial markets. Bitcoin as a financial instrument in the market is linked to the global economic conditions. Increased economic uncertainty encourages global investors to invest in Bitcoin, which is perceived as a safe asset. Bouri et al. (2017, 2018) mention that Bitcoin safeguards against policy uncertainty and serves as a safe-haven asset in times of financial turmoil. Demir et al. (2018) and Yen and Cheng (2021) explore the impacts of EPU on Bitcoin prices and find evidence that positive changes in EPU negatively affect Bitcoin returns. Cheng and Yen (2020) examine the interrelationship between the cryptocurrency market and EPU and find that EPU can influence the Bitcoin market. Shaikh (2020), using the quantile regression and Markov switching method on data from the U.S., the U.K., Japan, China, and Hong Kong, reveals that EPU affects Bitcoin returns. Besides, a study on the spillover effects of EPU on cryptocurrency markets shows that EPU can predict the cryptocurrency given the evidence of a strong interdependence (Foglia and Dai, 2021). Mokni (2021) finds that EPU affects Bitcoin volatility when the market is bullish and normal. Umar et al. (2021) examine whether Bitcoin can hedge against EPU and assert that U.S. investors can hedge Bitcoin against uncertainties. Li et al. (2022) decipher the volatility correlation between Bitcoin, stock, and gold in the context of uncertainty. The authors find that Bitcoin is positively correlated with high levels of EPU. In the context of the USA and China, Nasreen et al. (2022) explore the relationship between Bitcoin and EPU and find a positive return interdependence between them in the short run. The interaction suggests that frequent policy interventions link financial markets and EPU through which Bitcoin interacts. Therefore, the present study hypothesizes that economic policy uncertainty affects Bitcoin.

## 4 Methods

### 4.1 Wavelet coherence

The wavelet coherence is a popular method to study the co-movements and inter-shocks between a pair of time series. It captures co-movements between a pair of time series data over time and frequency scales. Thus, this method is suitable and relevant for financial markets as the participants in the cryptocurrency markets often trade at different times and scales, as reflected in their heterogenous time and investment horizons. Therefore, inter- and intra-market shocks can transfer from high to low frequency or vice versa, which provide essential information for investment decisions. According to Torrence and Compo (1998), two series b(t) and c(t) could explain the cross-wavelet transform in time sequence as:

(1) $$W_{bc}(m,n)=W_{b}(m,n)W_{c}^{⁎}(m,n)$$

In Eq. (1), $W_{b}(m,n)$ and $W_{c}(m,n)$ denote two continuous transforms of b(t) and c(t), respectively, m and n represent the position and scale, while the asterisk (*) indicates the composite conjugate. The value of $W_{bc}(m,n)$ shows the intensity of the correlation between b(t) and c(t). To show local covariance of two transformations between a pair of time series at each frequency, the study employs the modified wavelet coherence (Torrence and Webster, 1998) as:

(2) W2(m,n)=|S(n−1Wbc(m,n))|2S(n−1|Wb(m,n)|2)S(n−1|Wc(m,n)|2)

In Eq. (2) ‘*S*’ is a smoothing factor. The coefficient value of the $W^{2}$ is between 0 and 1. Smaller values indicate a weak correlation, while greater values show a strong correlation.

However, the value of $W^{2}$ is limited to positive values only, and thus, it cannot differentiate between positive or negative correlations. Therefore, the present study employs the wavelet phase difference (Torrence and Compo, 1998) to reveal the positive or negative correlation. The phase difference is specified as:

(3) ψb,c(m,n)=tan−1⁡(J{S(q−1Wxy(m,n))}L{S(n−1Wxy(m,n))}

In Eq. (3) *J* and *L* are the imaginary and real portion of the adjusted cross-wavelet transforms.

### 4.2 Toda-Yamamoto Granger causality

In the case of integration or cointegration, the traditional F-statistic from the application of the Granger causality test (Granger, 1969) can lead to spurious causality results. To address this shortcoming, Toda and Yamamoto (1995) use a modified Wald test statistic to examine Granger causality within a vector autoregressive (VAR) model. This paper performs the Toda-Yamamoto (T-Y) Granger causality test to uncover the causal relationship between the variables under study. It is specified as:

(4) $$X_{t}=\alpha_{0}+\sum_{f=1}^{k}\alpha_{1f}X_{t-f}+\sum_{j=k+1}^{d_{\max}}\alpha_{2j}Y_{t-j}+\sum_{f=1}^{k}\phi_{1f}X_{t-f}+\sum_{j=k+1}^{d_{\max}}\phi_{2j}Y_{t-j}+u_{1t}$$

and

(5) $$Y_{t}=\beta_{0}+\sum_{f=1}^{k}\beta_{1f}Y_{t-f}+\sum_{j=k+1}^{d_{\max}}\beta_{2j}Y_{t-j}+\sum_{f=1}^{k}\phi_{1f}X_{t-f}+\sum_{j=k+1}^{d_{\max}}\phi_{2j}Y_{t-j}+u_{2t}$$

In Eq. (4) and (5), *k* is the optimal lag length, $d_{\max}$ is the maximum order of integration. $Y_{t}$ and $X_{t}$ are two stationary variables. $u_{1t}$ and $u_{2t}$ are error terms.

The test is performed at VAR(p), where p is the optimal lag order plus $d_{\max}$ (maximum order of integration). First, the study first tests each time series by applying the ADF test and cross-checking it with the Phillips-Perron (P.P.) test to decide the maximum lag order. Then, the study uses the Akaike Information Criteria to determine the optimal lag order. Finally, the T-Y Granger causality test is performed.

### 4.3 Nonlinear Granger causality

The nonlinear and nonstationary properties of a volatile price series, particularly Bitcoin prices, need to be analysed by nonlinear methods. As the linear causality tests (here, T-Y) cannot account for nonlinear causality between the time series under study (Brock et al., 1991), the present study employs the nonlinear Granger test (e.g., Diks and Panchenko, 2006; Bekiros and Diks, 2008). To validate the use of the nonlinear Granger causality test, this study applies the linearity test (Brock et al., 1996), which enables the detection of deviations from independence in time series, i.e., nonlinear dependence. Then, the study performs the nonlinear Granger causality test following Diks and Panchenko (D-P) (2006). The null hypothesis of no Granger causality is specified as follows:

(6) $$Ho:\;Y_{t+1}|X_{t}^{\ell X};Y_{t}^{\ell Y}∼Y_{t+1}|Y_{t}^{\ell Y}$$

In Eq. (6) $X_{t}^{l_{x}}=X_{t+1},\ldots ,\;X_{t}$ and $Y_{t}^{l_{y}}=Y_{t+1},\ldots ,Y_{t}$ are the delay vectors and ($l_{x},l_{y}=1$) are the lag length of $X_{t}$ and $Y_{t}$. The ‘∼’ denotes symmetric distribution. Assuming $Z_{t}=Y_{t+1}$ and ($\ell_{X}=\ell_{Y}=1$), the joint probability density function $f_{X,Y,Z}(x,y,z)$ of ($W_{t}=X_{t}^{\ell X},Y_{t}^{\ell X},Z_{t}$) can be obtained in the following relationship.

(7) fX,Y,Z(x,y,z)fY(y)=fX,Y(x,y)fY(y)⋅fY,Z(y,z)fY(y)

For each fixed value of *y*, *X* and *Z* are conditionally independent on Y=y, according to Eq. (7). Thus, the revised H_0_ is found using Eq. (8) as follows:

(8) $$q=E[f_{X,Y,Z}(X,Y,Z)f_{Y}(Y)-f_{X,Y}(X,Y)f_{Y,Z}(Y,Z)]=0$$

Let ${\overset{ˆ}{f}}_{w}W_{i}$ represent local density estimator of $d_{w}$-variate random vector W at $W_{i}$ defined by ${\overset{ˆ}{f}}_{w}W_{i}=\{{\left(2\varepsilon_{n)}\right)}^{-d_{w}}/(n-1)\}\sum_{jj\neq i}I_{ij}^{W}$, where $I_{ij}^{W}=I∥W_{i}-W_{j}∥<\varepsilon_{n}$, where indicator function I(⋅) and the bandwidth $\varepsilon_{n}$, depending on the sample size n. Finally, the test statistic can be formulated in terms of a sample version q using Eq. (9).

(9) Tn(εn)=nn(n−2)⋅∑1fˆX,Y,Z(Xi,Yi,Zi)fˆY(Yi)−fˆX,Y(Xi,Yi)fˆY,Z(Yi,Zi)

For ($\ell_{X}+\ell_{Y}=1$, if $\varepsilon_{n}=Cn^{-\beta }$ (C>0, $\frac{1}{4}<\beta <\frac{1}{3}$), the test statistic $T_{n}(\varepsilon_{n})$ satisfies:

(10) n=(Tn(εn)−q))Sn→DN(0,1)

The term ($\to^{D}$) denotes convergence in distribution and $S_{n}$ is an estimator of the asymptotic variance of $T_{n}(\cdot )$. The test statistics from Eq. (10) are applied to the VAR residuals. Because VAR models remove linear predictability, the remaining iterative predictive ability of one series for another implies nonlinear causality (Yu et al., 2015).

## 5 Results and discussion

### 5.1 Data and preliminary analysis

This study uses the monthly data of Bitcoin prices, M2 (M2 = cash, demand, and checkable deposits), inflation, and EPU. Monthly Bitcoin price data were downloaded from finance.yahoo.com,^1^ and monthly CPI and M2 data from Federal Reserve Economic Data (FRED).^2^ The EPU index, developed by Baker et al. (2016), is obtained from economicpolicyuncertainty.com.^3^ The sample period is 31 July 2010–30 August 2020, as dictated by data availability. All the series are log-transformed by applying the following formula $R_{t}=(\log R_{t}-\log R_{t-1})\times 100$, where $r_{t}$ is the log return in series *R*, at time *t*.

Table 1 presents the summary statistics of the transformed data series. The Jarque-Bera (J.B.) statistics reject the null hypothesis of normality. Furthermore, the results of Augmented Dickey-Fuller (ADF) and Phillips-Perron (P.P.) tests indicate the stationarity of the time series.

**Table 1:** Descriptive statistics.

Series	Mean	St. Dev.	Skewness	Kurtosis	JB	ADF	PP
BTC	4.327	15.051	1.714	8.520	212.91***	−8.563***	−8.547***
U.K. M2	0.352	11.923	0.256	2.844	1.448***	−8.780***	−48.256***
Japan M2	0.129	0.203	1.162	4.919	45.829***	−3.491***	−7.985***
U.K. inflation	0.070	0.138	−0.470	4.322	13.277***	−2.888***	−11.949***
Japan Inflation	0.025	0.146	3.203	26.062	288.62***	−8.079***	−8.044***
UK EPU	0.076	12.858	0.009	2.711	0.422***	−5.495***	−16.066***
Japan EPU	−0.041	8.246	−0.339	4.338	11.356***	−3.054***	−18.209***

*** p< .01. The sample period is 31 July – 30 August 2020.

### 5.2 Brock–Dechert–Scheinkma test

The study performs a nonlinearity test on the VAR residuals, using the Brock–Dechert–Scheinkma (BDS) test to examine if a series is nonlinearly dependent. Specifically, Brock et al. (1996) proposed the BDS test based on the correlation to check the nonlinear dependence of financial series data. While performing the test, we select dimension *m* from 2-6 and a distance (*ϵ*) of 0.7.

The results of the BDS test (Table 2) show the presence of nonlinearity in most of the time series, which confirms the precondition to applying nonlinear methods to examine the relationship between the variables under study.

**Table 2:** BDS test results.

Country	m	BDS Test Statistics			
		BTC	M2	Inflation	EPU
UK	2	0.0145**	0.0057*	0.3869**	0.0108**
	3	0.0279**	0.0091	0.7177*	0.0195**
	4	0.0432***	0.0109	0.7945*	0.0200**
	5	0.0479***	0.0114	0.9591	0.0241**
	6	0.0487***	0.0110	0.8028***	0.0202**

Japan	2	0.0145**	0.0059*	0.0080***	0.007***
	3	0.0279**	0.0283***	0.0087	0.0120**
	4	0.0432***	0.0193	0.0081	0.0143
	5	0.0479***	0.0106**	0.0172	0.0150**
	6	0.0487***	−0.0037	0.0220	0.0142**

*** p<.01,

** p<.05,

* p<.1.

### 5.3 Empirical results from wavelet coherence

**Figure 1:**
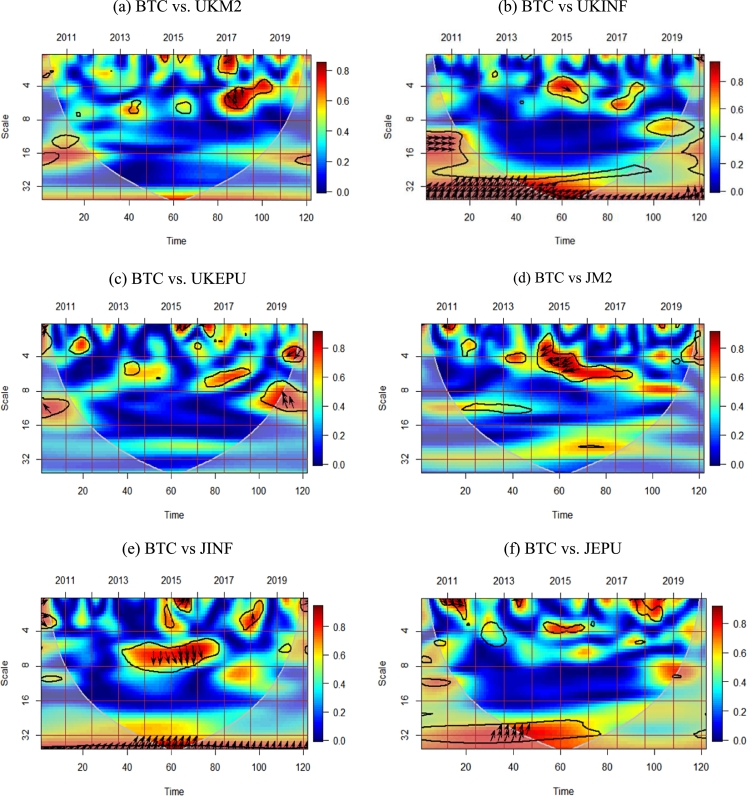
Wavelet coherence pairwise estimates. *Notes:* The black contour encircling red patches indicate the 5% significance level. The X-axis is each pair's timeline, while the Y-axis indicates the period (in months).

This research investigates the co-movement and causal relationships between the variables (BTC, M2, inflation, and EPU), using pairwise plots with wavelet coherence. The wavelet phase-discrepancy and coherence show cause-effect interactions between the variables under study. Fig. 1 shows the results plot- and pairwise.

**United Kingdom**

The wavelet coherence in Fig. 1(a) shows the interrelationship between the BTC and U.K.'s M2. The BTC-UKM2 pair shows a significant co-movement at a 2–8-month frequency in 2014-15, 2017-18, and 2020. The red area depicts the increased volatility of BTC in 2020 due to the COVID-19 pandemic. The arrows (left down) in 2017 mean that UKM2 was negatively correlated with BTC. A lower interest rate is generally associated with reduced demand deposits, which explains the negative correlation between BTC and M2. Thus, central banks can raise (reduce) interest rates to counteract Bitcoin's price effects. Fig. 1(b) shows the co-movement between BTC and inflation in the long run, where BTC positively affects inflation, also supported by (Conlon et al., 2021). It is reasonable to see that Bitcoin prices may affect inflation due to its currency property. In particular, Narayan et al. (2019) show that growth in Bitcoin prices is linked to inflation in Indonesia. In the short term, during 2020, BTC negatively affected inflation, while in 2015, inflation positively led to BTC. This finding suggests that inflation significantly affects Bitcoin's volatility in the long term, which revalidates Bitcoin's inflation-hedging properties. Fig. 1(c) shows a significant correlation between BTC and UKEPU in 2015–16 and 2019–20 at a 2–8- and 4–16-month scale, respectively. The arrows (right-up and left-down) in 2019–20 at a 2–4- and 8–16-month scale indicate that BTC and UKEPU are positively correlated in the short term, and negatively correlated in the medium term, which is similar to the findings of (Hernandez et al., 2021). The economic uncertainty in the U.K. helps increase investment in Bitcoin for its safe-haven properties. The results suggest that U.K. investors can carefully invest in Bitcoin depending on the time-lag effects, i.e., positive in the short term and negative in the medium term. Overall, BTC negatively leads to M2, while UKEPU positively leads BTC. BTC and inflation share a negative correlation in the short and long term.

**Japan**

The wavelet coherence between Bitcoin prices and Japan's M2 in Fig. 1(d) shows significant interdependence at a 2–8-month scale. The pair is out-phase (lower-left arrows) at 2014–2016, meaning Japan's M2 negatively leads BTC in the short term, while no long-term correlation exists. Short-term adverse effects could involve monetary policy implications, such as increasing interest rates (Marmora, 2022). This result signifies that increased Bitcoin investment may occur due to the increased money supply, which lowers the interest rate. During 2013–15, the interest rate in Japan dropped drastically from 1.664 to −0.948, which caused investors to divert their funds to a digital asset like Bitcoin. The correlation between BTC and inflation in Fig. 1(e) shows that BTC positively moves with inflation at a 16–32-month scale, indicating that inflationary pressure can cause Bitcoin prices to increase (Choi and Shin, 2022). The loss of value in savings deposits due to high inflation in fiat currency often motivates investors to invest more in an inflation-hedged asset like Bitcoin. Thus, the correlation between Bitcoin prices and inflation is positive. The right-up arrows in Fig. 1(f) show that BTC leads JEPU positively in the long term, implying that Japan's economic uncertainty is adjusted in the Bitcoin prices in the long run horizons. Overall, M2 negatively affects BTC in the short term, while BTC positively affects inflation and JEPU in Japan.

### 5.4 Evidence from Toda-Yamamoto Granger causality

The Toda-Yamamoto Granger causality test results (Table 3) show that M2 has a unidirectional causal impact on BTC in the U.K., while BTC shows no causal effect on the U.K.'s M2. This can be explained by the difference in interest rates on demand and time deposits. When the central banks increase interest rates, many risk-averse investors may divert their funds from risky investments such as Bitcoin to demand and time deposits. Notably, inflation and BTC have a bidirectional causality, meaning Bitcoin prices and inflation can help predict each other. As a hedging instrument, Bitcoin provides an opportunity to trade in tandem with inflation. Therefore, investors in the U.K. can hedge Bitcoin against inflation. Furthermore, both inflation and M2 have a bidirectional influence on each other, signifying the linear causal relationship between them. However, no causal relationship exists between EPU and the other variables in the U.K. In the case of Japan, M2 Granger causes BTC in Japan, and, unlike in the U.K., EPU has significant predictive power on BTC in Japan, which supports recent studies (Matkovskyy et al., 2020). Further results indicate that BTC influences EPU in Japan. However, no causal relationship exists between BTC and Japan's M2.

**Table 3:** Toda-Yamamoto Granger causality test results.

Country	*H*_o_	Test Statistics	P-value	Significance
U.K.	M2 → BTC	0.5534	0.0670	*
	INF→ BTC	10.5768	0.0050	***
	EPU→ BTC	1.6577	0.4365	
	BTC→ M2	0.1151	0.9441	
	INF→ M2	8.7378	0.0127	**
	EPU→ M2	4.2798	0.1177	
	BTC→ INF	2.0237	0.0035	**
	M2→ INF	7.7461	0.0208	**
	EPU→ INF	1.4624	0.4813	
	BTC → EPU	0.2892	0.8653	
	M2→ EPU	0.5616	0.7552	
	INF→ EPU	2.3689	0.3059	

Japan	M2→ BTC	6.4089	0.0093	***
	INF→ BTC	1.0455	0.7902	
	EPU → BTC	3.5941	0.0088	***
	BTC → M2	4.8098	0.1863	
	INF → M2	2.1091	0.5501	
	EPU → M2	2.0027	0.5718	
	BTC → INF	2.4379	0.4866	
	M2 → INF	14.8675	0.0019	***
	EPU→ INF	4.2798	0.0328	**
	BTC→ EPU	2.9896	0.3932	*
	M2→ EPU	0.8056	0.8481	
	INF →EPU	2.2047	0.5310	

*** p<.01,

** p<.05,

* p<.1.

### 5.5 Evidence from nonlinear Granger causality

The results of the nonlinear causality test (Table 4) show that BTC Granger causes M2 and inflation in the U.K., while inflation has nonlinear causal effects on BTC and EPU. This may happen due to the changes in inflation expectations. Given the inflationary pressure, an investor will look for inflation-hedged investment opportunities. As an inflation hedge against fiat currency, Bitcoin prices appreciate under inflationary pressure and lowered interest rate conditions. The causal links between Bitcoin prices and inflation are revalidated by recent evidence (Zhu et al., 2017; Blau et al., 2021; Conlon et al., 2021). In addition to existing evidence of linear and nonlinear causal effects of inflation on BTC, our findings indicate that BTC Granger causes inflation in Japan. Thus, U.K. investors can use this finding for inflation hedging. Further results show that EPU has a significant nonlinear causal effect on BTC, supported by most recent studies (Choi and Shin, 2022; Mokni et al., 2021; Matkovskyy et al., 2020).

**Table 4:** Nonlinear Granger causality test results.

Country	*H*_o_	Test Statistics	P-value	Significance
U.K.	M2 → BTC	−0.206	0.0817	*
	INF→ BTC	0.920	0.0262	**
	EPU→ BTC	0.375	0.0015	***
	BTC→ M2	1.173	0.0796	*
	INF→ M2	0.121	0.5482	
	EPU→ M2	0.353	0.3775	
	BTC→ INF	0.066	0.5262	
	M2→ INF	0.281	0.3891	
	EPU→ INF	0.495	0.0407	*
	BTC → EPU	0.093	0.3537	
	M2→ EPU	0.053	0.5212	
	INF→ EPU	0.832	0.0116	**

Japan	M2→ BTC	0.469	0.5335	
	INF→ BTC	0.726	0.0409	**
	EPU → BTC	1.827	0.0338	**
	BTC → M2	−0.084	0.0388	*
	INF → M2	0.379	0.3522	
	EPU → M2	−0.817	0.7929	
	BTC → INF	0.35	0.0722	*
	M2 → INF	0.116	0.0312	**
	EPU→ INF	0.468	0.0197	**
	BTC→ EPU	0.724	0.2346	
	M2→ EPU	0.032	0.4871	
	INF →EPU	0.299	0.3825	

*** p<0.01,

** p<.05,

* p<.1.

The results show a new finding that Bitcoin prices nonlinearly Granger causes M2 in the U.K., revealing a unidirectional causality. This effect can be explained by the growing use of Bitcoin, which involves daily transactions in general, and trade and investment in particular. Bitcoin's utilization as a currency or a financial asset increases the transaction frequency, replacing the British pound sterling (GBP). The higher frequency of Bitcoin transactions affects the velocity of fiat currency, i.e., GBP, which can influence M2 in the U.K. Narayan et al. (2019) find that Bitcoin price change negatively influences the velocity of fiat currency which, in severe cases, can lead to a decrease in the M2. Thus, Bitcoin can significantly involve currency substitution (Schilling and Uhlig, 2019; Marmora, 2021), which can affect M2. Our empirical findings corroborate their predictions and provide information about policy implications for investors and central banks. Remarkably, the nonlinear causal effects of inflation on BTC are significant in the U.K., supported by recent studies (Blau et al., 2021; Conlon et al., 2021). Notably, the results indicate a new finding that inflation Granger causes UKEPU. Inflation is an essential factor of economic stability. In the U.K., from 2010 to 2020, the inflation rates show an increasing trend. Higher inflation expectations can adversely affect investments, resulting in increased economic uncertainty. Thus, inflation can affect UKEPU. The nonlinear causality shows that inflation has significant causal effects on Bitcoin prices in Japan. Due to Bitcoin's currency and inflation-hedging properties, investors and users often consider Bitcoin in inflationary periods, thus establishing a causal link between Bitcoin prices and inflation. Therefore, any upward change in inflation can involve simultaneous interaction with Bitcoin. BTC also Granger causes inflation in Japan. Thus, BTC and inflation have bidirectional Granger causality in Japan, meaning that BTC and inflation are interdependent, and inter-shocks are instantly transmissible between them. Recent studies (Marmora, 2021; Rastogi and Kanoujiya, 2022) support this finding. Investors and policymakers can utilize this finding to manage inflationary ramifications in tandem with Bitcoin prices. Besides, a nonlinear causal relationship shows that EPU has significant predictive power on BTC in the U.K. and Japan. Indeed, uncertainties about the decisions of governments and regulatory authorities decrease investors' trust in mainstream currencies and/or the entire economy, especially after the global financial crisis of 2008–9. This was also the time when Bitcoin was introduced to the markets. Therefore, by its nature, Bitcoin and other digital currencies are decentralized, secure alternatives to fiat currencies, especially during economic and geopolitical unrest. Therefore, the changes in the EPU index can affect Bitcoin prices. Moreover, BTC has a unidirectional nonlinear causality with M2, which empirically complements previous findings (Narayan et al., 2019).

### 5.6 Diagnostic tests

To validate the robustness of the models employed for Granger causality tests, the current study performs the heteroskedasticity, serial correlation, and normality tests on the VAR residuals.

Table 5 shows the diagnostic test results. The ARCH test statistics show that the residuals have equal mean-variant (homoscedastic) linear and nonlinear series. The Lagrange Multiplier (L.M.) test results show no serial correlation in the residuals. Jarque-Bera (J.B.) test results show that the residuals are normally distributed.

**Table 5:** Diagnostic test results.

	Toda-Yamamoto Granger causality			Nonlinear Granger causality		
Country	ARCH	LM	JB	ARCH	LM	JB
UK	127.781	18.949	155.921	182.501	15.054	228.767
p-value	(0.971)	(0.2715)	(0.163)	(0.107)	(0.890)	(0.520)
Japan	256.495	18.530 (0.294)	2290.201	167.618	7.156	3126.33
p-value	(0.221)		(0.880)	(0.3240)	(0.970)	(0.864)

*Note.* P-values in parentheses are significant at the 0.05 level.

## 6 Conclusion and policy implications

The paper conducts the wavelet-coherence analysis to understand the co-movements and causal relationships between Bitcoin and M2, inflation, and EPU in time horizons and frequency scales in two major economies, i.e., the U.K. and Japan. It further examines the linear and nonlinear Granger causality between them.

The wavelet coherence results show that Bitcoin affects M2 and correlates with inflation and EPU in both countries. Inflation positively affects BTC in the short term, which provides new information to Japanese investors, e.g., they can invest in Bitcoin under inflationary pressure. Bitcoin price negatively affects M2, while EPU positively leads BTC in the U.K. and Japan in the short term. The T-Y causality results reveal a bidirectional causality transmission between BTC, inflation, and EPU in the U.K. and Japan. This finding implies that EPU and inflation can predict BTC. In addition, UKM2 and JM2 Granger cause Bitcoin prices. This result provides important policy insights into the regulation of Bitcoin and M2 for monetary authorities. Further evidence shows a nonlinear bidirectional causality running from Bitcoin prices to inflation and a unidirectional causality running to the M2 in both countries. These findings indicate investment implications for investors for any expected change in inflation to affect the portfolio. Specifically, EPU shows a causal effect on BTC in Japan.

The above results provide detailed knowledge about the price behavior of Bitcoin in relation to M2, inflation, and EPU, which can be useful for making investment and policy decisions. The dynamic co-movement can be further rewarding for investors during the COVID-19 pandemic because knowledge of these dynamic interrelationships enables investors to harness gains from increased volatility in the Bitcoin markets and abnormal gains while leveraging the risks. Thus, our findings offer practical intuition about the dynamic co-movement and causal relationship between Bitcoin and inflation and EPU, which investors can use while making decisions regarding hedging and managing portfolios. The findings are also relevant to the policymaking of central banks and to proactively containing Bitcoin's price effects. Central banks can use this new information to monitor Bitcoin's dynamics and develop policy accordingly. Therefore, the insights achieved from the U.K. and Japan have crucial policy submissions for policymaking in countries that allows Bitcoin.

However, as the study concentrates on the U.K. and Japan, future studies may enrich the current literature by exploring Bitcoin's interactions with exchange rates, policy rates, trade policy, and interest rates in emerging economies.

## Declarations

### Author contribution statement

**Provash Kumer Sarker:** Conceived and designed the experiments; Performed the experiments; Analyzed and interpreted the data; Contributed reagents, materials, analysis tools or data; Wrote the paper. **Lei Wang, PhD:** Conceived and designed the experiments; Analyzed and interpreted the data; Wrote the paper.

### Funding statement

This research did not receive any specific grant from funding agencies in the public, commercial, or not-for-profit sectors.

### Data availability statement

Data will be made available on request.

### Declaration of interests statement

The authors declare no conflict of interest.

### Additional information

No additional information is available for this paper.

## Footnotes

1 www.finance.yahoo.com.

2 https://fred.stlouisfed.org.

3 www.economicpolicyuncertainty.com.
